# Exploring the use of gender‐inclusive language amongst health care students and staff in obstetrics and gynaecology

**DOI:** 10.1111/medu.70232

**Published:** 2026-04-27

**Authors:** Debbie Aitken, Georgia Lin, Bhabesh San San Wal, Merryn Rhodes, Mariam Aly, Jack Amiry

**Affiliations:** ^1^ Department of Education University of Oxford Oxford UK; ^2^ Harris Manchester College University of Oxford Oxford UK; ^3^ Brasenose College University of Oxford Oxford UK; ^4^ Nuffield Department of Women's and Reproductive Health University of Oxford Oxford UK; ^5^ Frimley Health NHS Foundation Trust Surrey UK; ^6^ Oxford Health NHS Foundation Trust Oxford UK; ^7^ Medical Sciences Division University of Oxford Oxford UK; ^8^ Pembroke College University of Oxford Oxford UK; ^9^ Royal Berkshire NHS Foundation Trust Reading UK

## Abstract

**Introduction:**

Gender‐inclusive language is increasingly recognised as essential in health care to ensure respectful and equitable care for transgender and gender‐diverse individuals. However, the adoption of gender‐inclusive language in Obstetrics and Gynaecology (O&G) may vary across generations and hierarchical levels, and the perspectives of students and staff on its use remain underexamined. This study aimed to explore how O&G learners and clinicians understand and use gender‐inclusive language.

**Methods:**

An exploratory qualitative study was conducted in a UK teaching hospital's O&G department. Data were gathered via an online survey (27 respondents) and follow‐up semi‐structured interviews (12 participants: 7 students and 5 clinical staff). The study design was guided by queer and generational theory, applied via a constructivist lens. Reflexive thematic analysis was used to identify key themes.

**Results:**

Four themes were generated, reflecting generational, educational and power dynamics in gender‐inclusive language usage. Students generally reported greater familiarity and commitment to gender‐inclusive language, whereas some senior staff, but also a minority of students, voiced reservations or confusion. Hierarchical barriers were noted, with students hesitant to challenge non‐inclusive language used by superiors. Participants highlighted perceived curricular gaps and limited explicit teaching on caring for sexual and gender minority patients, recommending longitudinal, practical training, supportive correction of mistakes and visible role‐modelling. Structural obstacles, such as electronic record systems lacking non‐binary options, further constrained inclusive practice. Despite varied enthusiasm, participants universally emphasised respectful communication as a common professional value, converging on the importance of inclusive language for patient dignity.

**Conclusions:**

Gender‐inclusive language uptake in O&G is shaped by generational, hierarchical, educational and structural factors; however, a shared commitment to respectful care provides common ground. Fostering supportive intergenerational dialogue and addressing structural barriers can enable consistent, respectful practice. Senior role‐modelling and structured opportunities for juniors to teach upwards may further shift clinical culture without overburdening minority staff, ultimately improving care for gender‐diverse patients.

## Abstract 2

Do students and O&G clinicians differ in using gender‐inclusive language? Supportive intergenerational dialogue and addressing barriers can enable consistent, respectful practice. #MedEd

## 1 INTRODUCTION

The use of gender‐inclusive language^1^ in health care is increasingly recognised as essential for ensuring equitable and respectful care for LGBTQIA+ individuals. We conceptualise gender‐inclusive language as a component of person‐centred care, understood as communications that avoid discriminating against a particular sex, gender or gender identity and do not perpetuate gender stereotypes. Gender‐inclusive language is particularly salient within Obstetrics and Gynaecology (O&G), a specialty historically framed as ‘women's health’ and characterised by frequent reference to sexed bodies, reproductive anatomy and intimate examinations.^2^ We use the terms ‘birth sex’ to refer to an individual's assigned sex at birth, usually set through clinician judgement based on genital anatomy or other biological attributes, and the term ‘gender’ as an individual's internal sense of self and social identity. Routine clinical encounters involve discussions of pregnancy, menstruation, lactation and genital anatomy, all of which require precise terminology while also carrying strong gendered assumptions. For transgender and gender‐diverse patients, such encounters may therefore heighten the impact of language choices, making O&G a critical setting in which to explore how gender‐inclusive language is understood, modelled and contested in practice.

This growing attention to language is part of a broader movement to expand traditional conceptions of women's health to include the diverse identities of people who access gynaecological, reproductive and perinatal services.^2^ Unconscious bias and discriminatory behaviour towards gender diverse individuals have been shown to contribute to negative health outcomes.^3^ Furthermore, gender diverse individuals can experience challenges engaging with health care services, in part due to experiences of discrimination and insensitive treatment.^4^ Studies also highlight the health disparities faced by marginalised or minoritised patients, such as the markedly higher mortality and morbidity experienced by Black and Asian people in pregnancy and the puerperium.^5^ Consequently, discussions of gender‐inclusive practice should address coexisting racial and ethnic disparities that remain pronounced in perinatal outcomes and acknowledge that gender identity intersects with race and ethnicity to shape access, experience and outcomes. In response to calls for more inclusive practice, organisations such as the Society for Maternal‐Fetal Medicine have formally committed to gender‐inclusive terminology in clinical research and care.^6^

Despite this momentum, implementation remains inconsistent across health care systems and generations. In both North America and the United Kingdom, younger clinicians—particularly those in obstetrics—have increasingly adopted terms like ‘gestational parent’, whereas many senior practitioners continue to rely on cisnormative frameworks.^7^, ^8^ A cisnormative framework centres the cisgender, heterosexual experience and positions cisgender experiences as ‘normative’ and thus measures everyone else *against* this expectation. This divide reflects uneven exposure to LGBTQIA+‐inclusive medical education: Recent graduates more often report receiving such training, whereas senior clinicians rely on less inclusive protocols.^9^, ^10^ A UK‐based study found that only 21% of senior consultants felt confident using gender‐inclusive language, compared to 68% of junior trainees who had experienced recent curricular reforms.^11^ The prevalence of generational differences has also been studied in other areas of medicine, such as surgery, mental health, and generational stereotypes and diversity in training.^12^, ^13^, ^14^, ^15^

Although UK training frameworks have evolved, including NHS initiatives and updates aligned with the Gender Recognition Act of 2004, gaps persist. Patients still report long waiting lists for gender‐affirming care and frequent misgendering in routine care; for example, the NHS Northern Region Gender Dysphoria Service reports an average wait of 6 years and 8 months for an initial assessment.^16^, ^17^, ^18^, ^19^ In this context, ‘gender‐affirming care’ encompasses the clinical, organisational and interpersonal practices that support transgender and gender‐diverse patients to receive appropriate, respectful health care.^4^ Gender‐inclusive language, by contrast, forms an important communicative component of such care, but more as a crucial component of *effective* gender‐affirming care. Institutional efforts, such as mandated training and inclusive population screenings, have yet to consistently change front‐line clinical behaviour.^20^

Underlying many of these issues is a persistent gap in formal education. A recent curricular audit showed that foundational subjects like anatomy and obstetrics still rely on outdated, binary‐based language that erases intersex and transgender experiences.^21^ Research suggests that increasing medical students' exposure to inclusive training improves both confidence and clinical attitudes, particularly when students are taught to use correct pronouns and avoid heteronormative assumptions in sexual health histories.^22^ However, senior staff often lack equivalent training, and the importance of mentorship or visible LGBTQIA+ role models in shaping clinical culture is frequently underappreciated.^23^ Numerous studies on best teaching practices for younger generations in medicine exist, such as the experiences of Generation Z, but few address the gaps in diversity, equity and inclusion knowledge across generations of medical professionals.^24^, ^25^

Although there is modest evidence that LGBTQIA+ training interventions improve knowledge and attitudes in the short term, little is known about how these reforms are perceived or implemented in practice, particularly by different generations of medical staff. Existing studies rarely explore the deeper cultural, institutional and interpersonal dynamics that shape how language is used or resisted in clinical environments. We posit that it is the role of health care providers to address this inequality and consider the influence of their own behaviour upon diverse patient groups.

This study aims to address that gap by exploring how gender‐inclusive language is understood, experienced and operationalised across generations of medical professionals, from students to senior practitioners, in O&G. Through an online survey and in‐depth semi‐structured qualitative interviews, we hoped to examine how inclusive practices may be taught, modelled or contested across professional hierarchies—and what these findings suggest about the broader institutional readiness for gender‐inclusive reform.

## 2 METHODS

### 2.1 Design and theoretical framework

We adopted an exploratory qualitative design to examine the use and understanding of gender‐inclusive language in undergraduate O&G education. Our approach was guided by queer theory and generational theory, employing a constructivist lens to craft interview questions and the analytical process. Queer theory, first introduced in the early 1990s as an expansion of lesbian, gay and gender studies, seeks to challenge the cisgender, heteronormative binaries that structure societal norms through a ‘queering’ of assumed identities.^26^ In medical education, enacting queer theory can involve adopting inclusive terminology for transgender and gender‐diverse patients and students to disrupt established conventions in medicine, whereby all patients are assumed to be cisgender and heterosexual.^27^, ^28^ Generational theory informed our consideration of how perspectives on gender‐inclusive language may vary across training stages and years since qualification, recognising that historically contingent exposures might influence attitudes towards inclusive language or knowledge gaps about sexual and gender minority (SGM) identities.^29^ Although the concept of a queer generational theory in combination has not been extensively studied, both frameworks influenced the development of our research questions and the interpretation of results.

Recognising that generational framings can inadvertently reproduce stereotypes or deficit assumptions, we treated generation as a sensitising lens rather than a deterministic explanatory variable. Drawing on the concept of ‘generational situatedness’,^14^ we interpreted apparent generational differences as shaped by historically contingent exposures (e.g., curricular reforms, sociopolitical discourse and team cultures) and attended to within‐group variation and intersections with professional hierarchy. We also attended to instances where participants themselves resisted simplistic generational narratives, using these reflections to guard against linear ‘progress’ framings in our interpretation.

### 2.2 Setting and participants

The study was conducted in the O&G department of a UK tertiary teaching hospital within a large NHS Foundation Trust, affiliated with a research‐intensive medical school. The department serves as a core clinical placement site for undergraduate medical students and operates within a busy service environment characterised by the competing demands of patient care and workplace‐based teaching. The setting is urban and serves a socioeconomically and demographically diverse population. As in many UK clinical training environments, teaching takes place within the broader context of NHS workforce pressures and time‐constrained service delivery.

**FIGURE 1:**
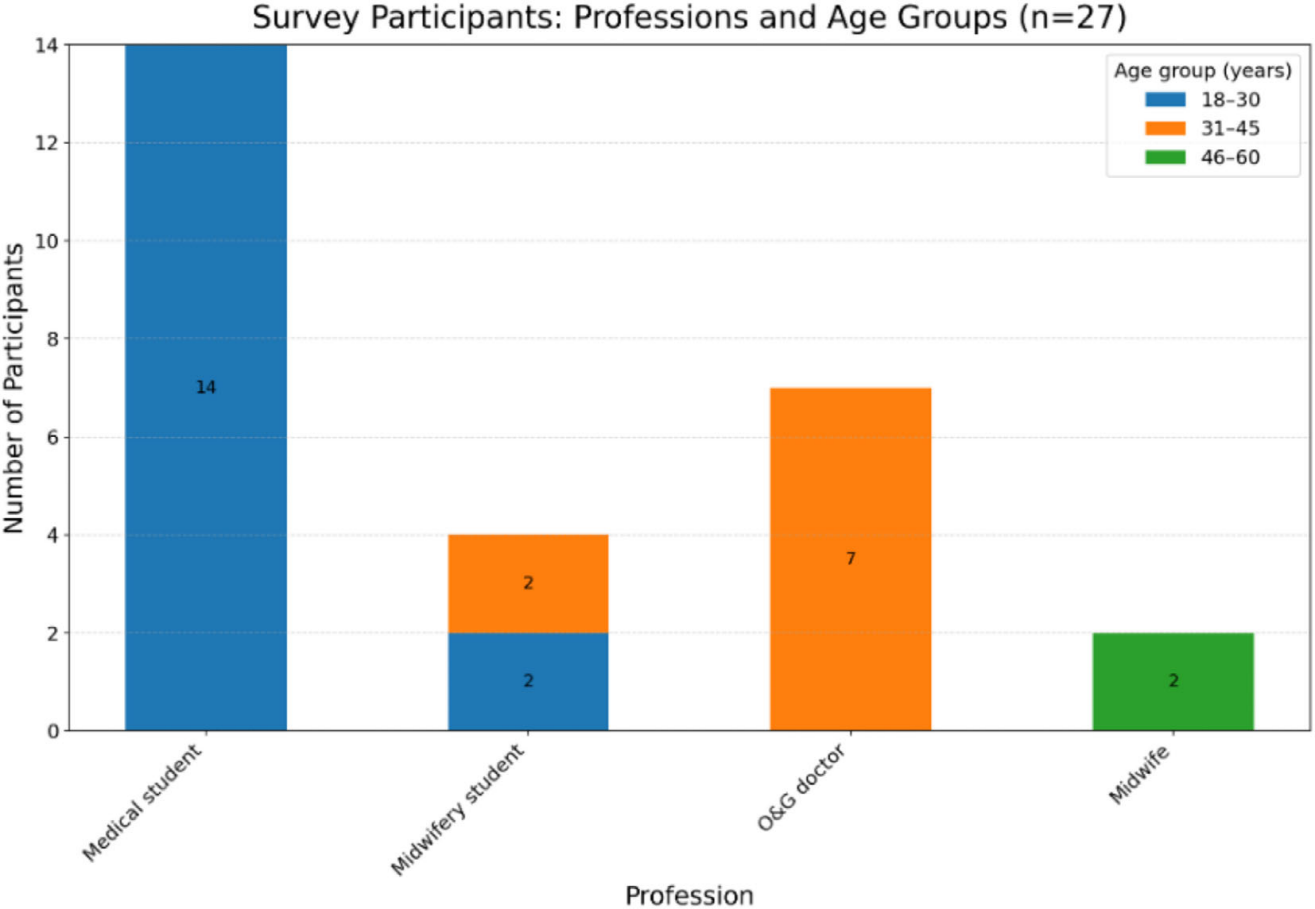
Survey participants by profession and age group. [Color figure can be viewed at wileyonlinelibrary.com]

**FIGURE 2:**
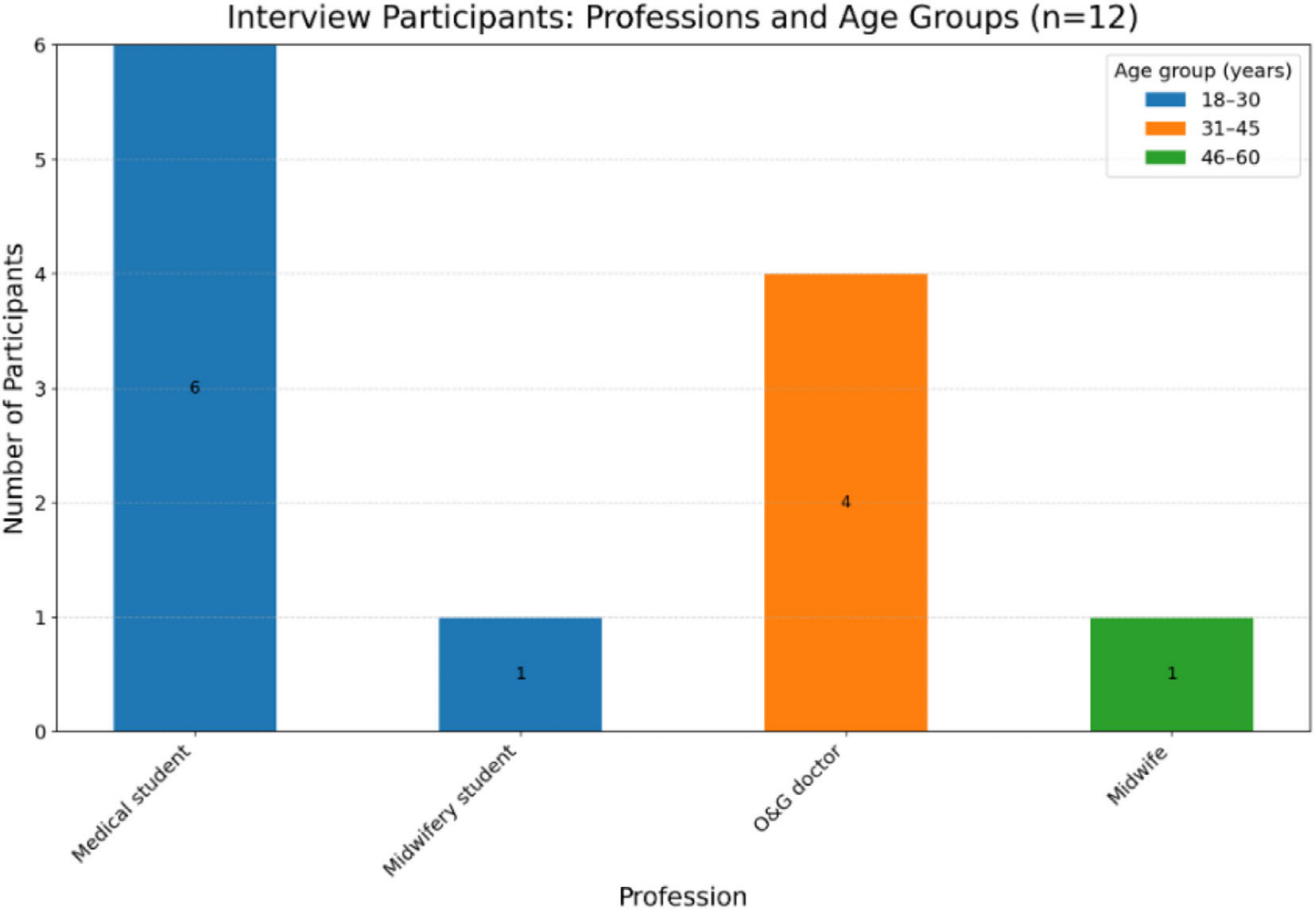
Interview participants by profession and age group. [Color figure can be viewed at wileyonlinelibrary.com]

Participants were recruited from two groups to explore perspectives across training stages and professional roles: medical and midwifery students undertaking O&G rotations and O&G clinical staff involved in teaching or supervising students during placements. Purposive sampling was used to include a range of roles and experiences within each cohort. Recruitment occurred via course announcements, departmental staff emails and promotion through medical student societies. An initial online survey, structured around two case studies developed by clinical staff on the research team, was completed by 27 respondents (see Figure 1). Of these, 12 individuals consented to follow‐up and participated in semi‐structured interviews to capture rich, contextualised accounts of language use and learning in practice (see Figure 2), resulting in seven student and five clinical staff interviews.

Data collection occurred within a broader institutional and sociopolitical context characterised by heightened public and professional scrutiny of equity, inclusion and discrimination in health care. Nationally, debates surrounding sex, gender identity and transgender health care in the United Kingdom had become increasingly polarised during the study period. Locally, the Trust had been subject to external review and public discussion regarding racism and discriminatory practices within clinical services and organisational culture. Although this study did not aim to evaluate institutional policies or initiatives, these contextual factors form an important backdrop to participants' accounts.

### 2.3 Data collection

**FIGURE 3:**
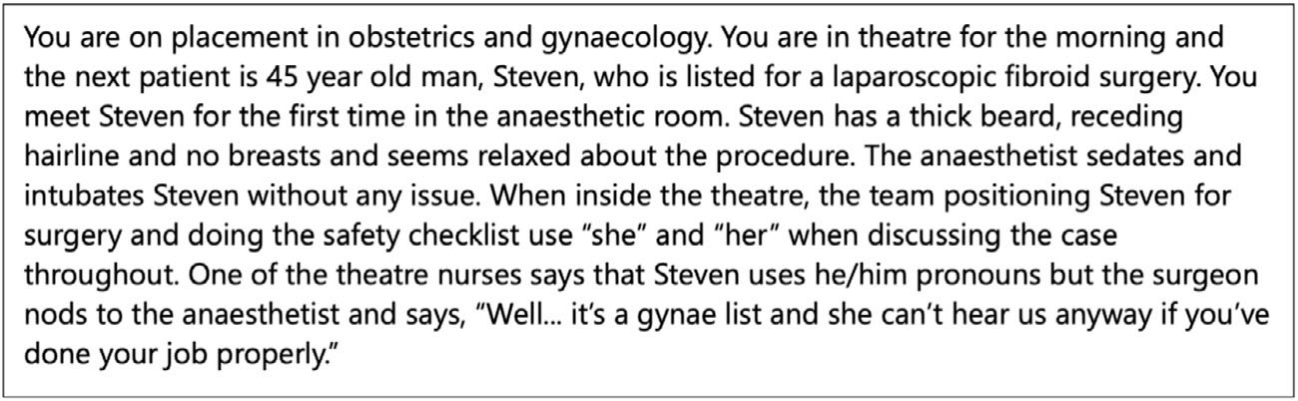
Scenario presented in online survey.

Data were collected in two stages, beginning with the online survey (Supporting Information S1) that gathered perspectives on gender‐inclusive language. The survey included demographic items (age range and role) and open‐ended questions inviting participants to reflect on a specific scenario presented in a case study, which was developed by the clinical staff on our team (see Figure 3).

In the second stage, we conducted in‐depth semi‐structured interviews with a subset of survey respondents who volunteered for further participation. We developed an interview guide (Supporting Information S2) informed by relevant literature and preliminary survey findings, focusing on how participants interpret, use and respond to gendered language in clinical teaching and practice. Interviews—lasting approximately 60 minutes—were conducted one‐to‐one, either face‐to‐face in a private setting or via a secure video platform (Microsoft Teams). All interviews were audio‐recorded with consent and transcribed verbatim, and identifying information was removed during transcription to ensure confidentiality. Participants were asked to choose a pseudonym for any quote attributions, a practice common in participatory research to encourage empowerment.^30^ For example, one participant asked for her pseudonym to be a female scientist, eventually choosing Marie Curie.

### 2.4 Data analysis

We used Braun and Clarke's^31^ reflexive thematic analysis to examine both survey and interview data. Our coding process was primarily inductive, allowing themes to be derived from the data, whereas our theoretical frameworks sensitised us to relevant issues—such as power dynamics in language and how experiences may differ across training and career stages. We analysed the combined dataset as a whole and used constant comparison to explore similarities, differences and disconfirming cases across participant roles, rather than treating age group as a deterministic explanatory category. We followed the standard phases of reflexive thematic analysis: familiarisation with the data, generation of initial codes, development and review of themes, and refinement of theme definitions. All transcripts and open‐ended responses were imported into NVivo 14 to facilitate coding and organisation.

Multiple researchers participated in the analysis. Two team members independently coded subsets of transcripts, developing preliminary codes. The team compared and consolidated these codes into a shared coding framework applied across the dataset. Multi‐researcher coding enhanced rigour: Analytic notes were added to transcripts and collectively reviewed to identify differing interpretations and biases. Codes were iteratively refined and clustered into candidate themes through constant comparison across data sources. Regular team discussions finalised themes, resolving divergent views through reflexive dialogue rather than mechanical consensus, ensuring the resulting themes authentically reflected participants' experiences.

To clarify our coding process and integration across data sources, analysis proceeded iteratively across both qualitative datasets (open‐ended survey responses to the vignette and interview transcripts). We first familiarised ourselves with the survey free‐text responses and generated initial codes inductively. These preliminary codes informed refinement of the interview topic guide and provided a starting coding framework (codebook) that was iteratively updated as new concepts emerged. Interview transcripts were then coded within NVivo using the evolving framework, with regular team discussions and reflexive memoing to refine code definitions and develop candidate themes. We used constant comparison to examine convergence, complementarity and disconfirming cases between survey and interview data, and final themes were developed to reflect patterns across the combined dataset.

### 2.5 Researcher reflexivity

Reflexivity was a continuous element throughout the study. The importance of recognising differing positionalities and its impacts have been documented in health care research.^32^, ^33^, ^34^ The research team included both senior and junior members working in educational studies, medical education, higher education and NHS clinicians, offering diverse generational and personal perspectives (more detailed reflexivity statements are provided in Supporting Information S3). We recognised that our backgrounds—professional roles, generational cohort and views on gender‐inclusive language—could influence the research. How our positionalities may influence power dynamics in relation to the interview participants, from vantages of an individual with perceived less power (e.g., a doctoral student interviewing a senior clinician) to seemingly parallel positionalities (e.g., clinicians interviewing clinicians) were key factors throughout the research process, including who would conduct each interview. To mitigate this, we engaged in reflexive practice at each stage.

For example, during data collection and analysis, we maintained reflexive journals and regular team discussions examining how assumptions or reactions shaped our questions or interpretations. This self‐reflection, consistent with our queer theory lens, helped us question normative biases and maintain critical awareness. Collaborative coding and annotation further facilitated reflexivity by highlighting different researchers' interpretations, which we discussed and critically examined together. By treating reflexivity as an ongoing stance rather than a discrete step, we enhanced the credibility and depth of our analysis.

### 2.6 Ethics

Ethical approval was obtained from the University of Oxford Department of Education Research Ethics Committee (Ref: EDUC_578331). Participants provided informed written consent after receiving study information. Participation was voluntary, with the right to withdraw until the project concluded with publication submissions. Confidentiality was strictly maintained; transcripts were pseudonymised, securely stored and contained no identifiable information. Participants had the opportunity to review their transcripts after the interview should they wish to make any changes. We recognised the potential power imbalances that could occur if the members of our team who worked in the medical school as clinicians were to actively promote participation in the project to their students. Therefore, recruitment was conducted through medical school‐wide mailing list announcements.

### 2.7 Limitations

As part of the survey questionnaire, respondents were asked to fill in their contact details so the research team could inquire further about interviews or their written responses. The inclusion of these details in the survey could have discouraged potential participants from responding given the sensitive nature of the subject; however, all participant literature emphasised the importance of providing honest responses for the project to gather holistic viewpoints on gender‐inclusive language and its uses in medical education. The data was collected at a single institution, with students and clinicians working within a single NHS trust; thus, their perspectives may not be representative of other medical trainees and professionals in other parts of the United Kingdom. In accordance with ethics, there exists no identifiable data that could lead to personal or professional consequences for participants exercising their freedom of speech.

## 3 RESULTS

The following results provide an overview of how gender‐inclusive language is operationalised in the clinical environment, directly addressing our aim to understand the perspectives of students and staff within O&G. By categorising findings into generational, linguistic and educational themes, we highlight not only the varying levels of familiarity with inclusive terminology but also the power dynamics that dictate whether such language is used in practice.

### 3.1 Themes and accompanying codes

We identified four main themes from the data analysis: *generational themes*, *language and inclusivity*, *clinical practice and education* and *intersectionality and trans rights*. The themes, accompanying codes and representative quotes are presented in Supporting Information S4.

### 3.2 Generational shifts

Participants sometimes framed their experiences in terms of ‘older’ versus ‘younger’ colleagues. We report these as participants' perceptions rather than a predefined ‘older generation’ category; for clarity, we describe participants by role and age range (Figures 1 and 2) and interpret differences in relation to training stage and years since qualification.

Overall, clinical medical students in training broadly favour using gender‐inclusive language across all medical specialities, not just O&G. New generations of medics have an existing knowledge of queerness and pronouns, allowing them to adapt faster when treating gender diverse patients. Though older generations of clinicians may not have learned gender‐inclusive language in their own medical training, Joy (clinician) reported the following:

Overwhelmingly my colleagues treat most people, there's no malicious intent, and I think that's really important. We can do better with pronouns … it doesn't really hurt us to call anyone however they want to be called. It literally takes no skin off your teeth.

Students reported apprehension and a fear of confronting established medical hierarchies, such as speaking up about senior staff for misgendering patients, as they were afraid it could have negative repercussions for their training. They also described witnessing discrimination against gender minorities by senior doctors, especially when clinicians were not in a patient‐facing environment and only speaking with colleagues. However, students and staff both mentioned positive markers of queer health care‐related cultural changes occurring in O&G, such as the normalisation of same‐sex couples conceiving.

### 3.3 Language and inclusivity

A central component of our study was to examine how the historical framing of O&G as ‘women's health’ influences current linguistic choices. All participants emphasised the importance of respect and dignity for patients, regardless of their gender identity, as a fundamental need in medical practice. Some clinicians expressed hesitation around officially changing titles such as ‘women's centres’ to be gender‐neutral due to gender diverse patients being a minority. There was also a pattern of people feeling that gender‐inclusive language is grammatically ‘clunky’, albeit a complaint that the majority of student respondents refuted as an insubstantial excuse to avoid using gender‐inclusive language altogether. For example, Edith (Y5 medical student) laid out her argument:

I think the sort of like minimum standard is if like, someone tells you their pronouns, use them. So (…) why is the hospital called the Women's Centre? I don't know. I feel like obstetrics and gynaecology unit is like good enough and it's a better reflection of what the service actually provides as well.

The nuance lies in respondents' varying attitudes towards individual changes—such as addressing a patient by their correct pronouns, for which they were all in favour—compared to systemic changes, such as amending official documentation or curricula to no longer specify ‘women's health’, which was seen as a more contentious and potentially harmful change from those who were more sceptical about gender‐inclusive health care policies.

### 3.4 Clinical practice and education

Implementing gender‐inclusive language in O&G clinical practice has mixed applications, especially as practitioners hold differing views about changing established linguistic conventions, for example, breastfeeding versus chest‐feeding, a person who menstruates or people with cervixes. Medical students felt they were not adequately taught how to treat SGM patients and expressed a desire for additional gender‐inclusive training across the curriculum, such as learning about available screening programmes for SGM patients, not just in O&G or reproductive health modules.

Participants also suggested that the uptake of gender‐inclusive language is shaped by the extent to which inclusive terminology is embedded into formal learning and institutional guidance. One clinician noted that when exam questions and clinical guidelines model gender‐inclusive language, this can help make such terminology routine in day‐to‐day practice:

So if the exam questions and the guidelines are using gender inclusive language, then that's the language we learn and therefore naturally that should fall into our day‐to‐day work. (Reu, Clinician)

Participants also described systemic constraints that can limit inclusive practice even when individual clinicians intend to document gender identity accurately. For example, one student highlighted that electronic systems often default to binary categories, meaning that additional gender identity information may not be visible unless clinicians actively search within the record:

There might be scenarios where on the computer system it will flash up as one of the patients is male or female. There's not usually an option to put any other gender identity or nuance in there, that you have to look through the notes to figure that out. (Joseph, Y6 medical student)

Both students and clinicians observed the changing nature of the health care profession, as Jane (midwife) noted:

What we also can't ignore is the minorities, the LGBTQ people, are coming into the profession as well. So, those that are educating our future professionals have to be aware that maybe these are the people we're educating as well, and so it has to run right through the centre of everything we do. So yeah, [queer healthcare] has a huge role to play and like I've said, hopefully it will just be eventually, it will just be who we are and what we teach, and it will just be as accepted as men and women are now.

As clinicians trained more recently start practising in O&G, respondents appreciated the need for the speciality to evolve with changing societal and gender norms.

### 3.5 Intersectionality and trans rights

Participants understood that there exists a volatile political climate around trans rights in the United Kingdom. At the time of data collection, a Supreme Court judgement ruled that the Equality Act refers only to ‘biological sex’ and therefore the definition of ‘woman’ excludes trans women, though it did not mention intersex individuals.^35^ The majority of students and clinicians surveyed were sympathetic to how transphobia may affect SGM patients accessing health care, with Matilda (Y5 medical student) laying out the potential risks:

I feel like the politics make it seem like kind of everyone's against trans people and then if a trans person is seeking healthcare, even for something completely unrelated, I feel like they would be less willing to do that and would kind of approach it feeling that people might not accept them or might misgender them or kind of things like that just because everything coming from above is like, ‘trans healthcare is bad’.

However, some respondents did express a concern that trans rights could usurp established women's rights, hence their apprehension around adopting gender‐inclusive language related systemic and curricular changes.

## 4 DISCUSSION

Discussions of gender diversity and gender‐inclusive language in medicine often risk becoming polarised. Readers may have been tempted to skip ahead to see whether we were ‘with them’ or ‘against them’. This underscores a symptom of the wider politicisation of sex and gender in the United Kingdom, in addition to the prevalence of transphobia in health care, where individuals could be encouraged to take sides rather than engage with complexity and nuance.^36^ Our findings suggest that progress is less about winning debates than about fostering respect, reflection and openness to learn from one another.

### 4.1 Hierarchy and generations

One of the clearest dynamics in our data was the role of hierarchy. Seniors not only hold authority in clinical decision‐making but also set the cultural tone of a team. In education, hierarchies function epistemologically as well as organisationally—shaping who has access to both formal and informal knowledge in medical settings. Participants highlighted a lack of training at formative stages, reinforcing the suggestions from the literature that adoption of inclusive terminology early in medical school is more likely to lead to lasting linguistic change.^22^ We argue that although hierarchies are useful in contexts such as acute clinical care, they should not extend unquestioned into domains such as language and identity. It would be reductive, however, to treat confidence with gender‐inclusive language as purely generational but rather in tandem with critiques of cisnormativity and heteronormativity in medicine. Scepticism towards gender‐inclusive language was voiced by student respondents, whereas several senior clinicians evidenced self‐reflective and adaptive practices for broader inclusion. Attitudes towards inclusive language therefore cut across age and experience, even if younger generations—ostensibly socialised with diverse pronouns and queer identities—may feel more comfortable. It is therefore worth asking why we might feel less comfortable inviting others to guide discussions around gender‐inclusive language in clinical settings.

Indeed, it is arguably unfair to expect senior clinicians to educate themselves to a point where they become the primary teachers of concepts absent from their own training. Allowing students and early‐career staff to take up some of this pedagogical role may redistribute responsibility more equitably, flattening hierarchies while relieving seniors of a burden for which they may feel ill‐equipped. It is crucial to also avoid the ‘minority tax’, where a disproportionate (often uncompensated) responsibility falls on underrepresented individuals to educate and guide their seniors.^37^ Yet the entrenched authority of senior clinicians can make such shifts difficult to enact in practice. Hierarchy in medical settings not only structures decision‐making but can generate intimidation, leaving junior staff or students reluctant to assume a pedagogical role even where they may have greater fluency with inclusive terminology.

The use of gender‐inclusive language is especially relevant within O&G due to the nature of the speciality, and, as observed by some participants, discussions and changes to adopt gender‐inclusive language have already begun within the field, which can also indicate a growing societal understanding of queerness.^38^ Guidelines now often refer to ‘women and birthing people’, and gradual acceptance of non‐typical pronoun use was noted by participants.^39^ Adapting terminologies to different populations was considered important by participants. Although the use of gender‐inclusive language may vary between the fields of O&G, misgendering patients could have an adverse effect on health outcomes in both.^40^

It is worth noting that, although much of our analysis considers hierarchy in terms of seniority and experience, we feel hierarchies between professional groups hold potential to shape language practices in O&G. Interprofessional relationships and different professional cultures, as well as differences in perceived authority between doctors, midwives and nurses, may influence who feels able to challenge or model inclusive language and whose corrections are taken up or resisted. These professional hierarchies may intersect with generational positioning, amplifying power differentials for some groups while mitigating them for others. Although our study was not designed to compare professions systematically, recognising these layers adds nuance to understanding how inclusive practices are enacted or contested within clinical teaching environments. This may therefore be a worthwhile area for further research.

### 4.2 Considering ‘the real world’

Although our findings point to broad principles for fostering inclusivity, we recognise that implementation will be shaped by the realities of clinical practice and institutional systems. Participants highlighted structural barriers—from IT systems that cannot accommodate non‐binary identities to the absence of safe, anonymous mechanisms for students to raise concerns—which risk undermining even the most well‐intentioned educational reforms. Furthermore, although our focus was on gender‐inclusive language, participants naturally drew connections to other forms of discrimination—including race, disability and sexuality. These intersections matter, as the experience of marginalisation in health care is rarely confined to a single axis of identity, and viewing gender‐inclusive language in isolation risks missing its role within broader cultures of equity and respect. Future research and curricular reform might therefore benefit from situating gender‐inclusive language within a wider equity framework, enabling health care professionals to see inclusivity not as piecemeal adjustments but as a holistic commitment to dignity across difference.

Within the wider context of medical misogyny, some participants' hesitancy was rooted as a concern that gender‐inclusive reforms risk eroding the identity of cisgender women and linked to concerns around systemic change or ‘clunky’ language.^41^ These tensions may prove polarising, particularly for those who have long identified O&G and midwifery as ‘women's health’. Yet it is important to emphasise that extending respect and recognition to trans and gender‐diverse individuals should not, *as a matter of principle*, diminish or threaten the rights of cisgender women. Framing the issue as a zero‐sum conflict risks obscuring the fact that everyone is negotiating the same patriarchal structures that have historically constrained identity and autonomy. Our findings therefore highlight the need for mindful, sensitive discussion that reassures all women their identities are respected while also ensuring the meaningful inclusion of gender‐diverse individuals.

This is reflected in our finding that ultimately, all participants converged on the language of respect. Advocates and sceptics alike were motivated by the desire to be a ‘good doctor’ or a ‘good midwife’, even when their enthusiasm for gender‐inclusive language varied. Although sceptical or dismissive remarks were noted frequently, these were often not intended as malicious but could still shape team culture and be experienced as harmful.^42^ This behaviour can especially impact gender‐diverse individuals who may not be ‘out’ (including within a health care team), making the impact difficult to quantify. Yet participants also pointed to precedents for change, noting how respectful terminology around sexual orientation has gradually become normalised in clinical contexts. This suggests that mindful engagement and institutional support can embed inclusive language in everyday practice, helping it become a routine marker of respectful care.

It is important to emphasise that our findings do not suggest that language alone is sufficient to constitute ‘gender‐affirming care’ as we conceive it. Rather, participants' reflections illustrate how communication practices operate alongside, and sometimes independently from, clinical decision‐making. As with other domains of person‐centred communication in medicine, inclusive language may not alter diagnostic reasoning or treatment algorithms but remains integral to how care is received and whether patients feel recognised within clinical encounters.

## 5 CONCLUSION

Gender‐diverse individuals continue to experience poorer health outcomes, often linked to discrimination and insensitive treatment, which in turn deters engagement with health care.^43^ It is important to note that although we used ‘gender diverse’ as our primary descriptor in this paper, we recognise that terminology in this space is fluid and contested nor is it definitive or immutable. Language in this field is often evolving rapidly as existing terms are weaponised by external actors or outpaced by the lived experiences of the community. Recognising these realities, professional organisations have formally committed to gender‐inclusive terminology as a standard of good practice. In training future generations of medical professionals, it is imperative that not only gender‐inclusive language but holistic, affirming care is placed at the forefront of medical education. Working towards a health care paradigm that recognises the need for solidarity across movements is vital to ensuring our collective liberation.

The challenge for health care educators is perhaps not only to emphasise how important inclusive language can be to those who it acknowledges and recognises but to understand that most people are doing their best within the context of their lived experiences and training. Crucially, this also means acknowledging the hierarchies that shape medicine and the perceived generational differences in knowledge and comfort with gender‐inclusive language. Combative confrontation risks defensiveness and division; supportive correction, dialogue and positive reinforcement were instead seen by participants as more likely to foster lasting change. If we are asking individuals to fundamentally re‐examine how they understand the people in their care, then the task is to create a supportive environment where mistakes, reflection and mutual teaching are possible. We believe that compassionate, respectful and non‐judgemental dialogue is not a soft option, but the most realistic route to shifting entrenched worldviews on questions as fundamental as identity.

## AUTHOR CONTRIBUTIONS

**Debbie Aitken:** Conceptualization; investigation; funding acquisition; writing—original draft; methodology; project administration; formal analysis; visualization; writing—review and editing; supervision; data curation. **Georgia Lin:** Investigation; project administration; formal analysis; data curation; methodology; visualization; writing—review and editing; writing—original draft. **Bhabesh San San Wal:** Formal analysis; investigation; writing—original draft; writing—review and editing; visualization. **Merryn Rhodes:** Formal analysis; investigation; writing—original draft. **Mariam Aly:** Formal analysis; investigation; writing—original draft. **Jack Amiry:** Investigation; funding acquisition; writing—original draft; formal analysis; visualization; writing – review and editing.

## CONFLICT OF INTEREST STATEMENT

The authors declare no conflicts of interest.

## ETHICS STATEMENT

This study was reviewed and approved by the University of Oxford Department of Education Research Ethics Committee (Ref: EDUC_578331). All participants provided informed written consent prior to participation.

## Supporting information


**Data S1.** Online survey instrument, including demographic items and case‐based questions.

**Data S2.** Semi‐structured interview guide used in data collection.

**Data S3.** Reflexivity statements from members of the research team.

**Data S4.** Themes, accompanying codes, and representative participant quotes from the analysis.

## Data Availability

The data that support the findings of this study are available on request from the corresponding author. The data are not publicly available due to privacy or ethical restrictions.
