# Supplementary material for: Exploring the use of gender‐inclusive language amongst health care students and staff in obstetrics and gynaecology
Source: Med Educ. 2026 Apr 27;60(10):1110–20. doi: 10.1111/medu.70232 (PMC13569165; doi:10.1111/medu.70232)
Supplement: Supplementary file 1 [file MEDU-60-1110-s001.docx]

# **Supplementary Material 1**

**SURVEY QUESTIONS**

**Section One**

1. Pronouns – if you wish to share them, (Pronouns are how people refer to you for example "she" or "her". Other examples include he/him, they/them, xe/xir and others.)
2. Age range
a. 18-30
b. 31-45
c. 46-60
d. 61-79
e. 80+
3. University affiliation (if any)
4. If you are a medical student, please choose your year of study (years 1-6).
5. If you are a member of clinical staff, please specify your general role in relation to education (E.g. pastoral support, assessment, clinical teaching, etc)
6. If you are a member of clinical staff, please specify your general role in relation to education (E.g. pastoral support, assessment, clinical teaching, etc)

**Section 2 – Research-Specific Questions** *(Multiple answers are acceptable)*

1. Have you interacted or treated any gender-diverse patients in your clinical training (or teaching)?
   a. Yes, in teaching
   b. Yes, in training
   c. No
   d. I am not sure
   e. Not to my knowledge
   f. It is impossible to know
2. Do you ask the pronouns of patients when you meet them?
a. Yes
b. No
c. Sometimes I do, sometimes I don’t
d. I don’t think it’s needed
e. I don’t feel I understand pronouns well enough
f. I don’t ask directly but leave a space for them to tell me
3. Are you interested in further participating in an interview with one of the research team members to learn more about your experience?
   a. Yes, in person
   b. Yes, online via MS Teams
   c. No

**Section 3 – Case Study Scenario** *(Text answers with no word count limit)*

You are on placement in obstetrics and gynaecology. You are in theatre for the morning and the next patient is 45yr old man, Steven, who is listed for a laparoscopic fibroid surgery. You meet Steven for the first time in the anaesthetic room. Steven has a thick beard, receding hairline and no breasts and seems relaxed about the procedure. The anaesthetist sedates and intubates Steven without any issue. When inside the theatre, the team positioning Steven for surgery and doing the safety checklist use “she” and “her” when discussing the case throughout. One of the theatre nurses says that Steven uses he/him pronouns but the surgeon nods to the anaesthetist and says “Well...it’s a gynae list and she can’t hear us anyway if you’ve done your job properly.”

1. Do you think there is any issue using she/her pronouns for Steven in this situation?
2. If you felt there was an issue, would you feel comfortable addressing it with the team? If so, would you do it in the moment or discuss it later? If you do not feel there is an issue, please outline your thoughts on the situation.
3. Would you feel differently if this was a high-level emergency such as a Category 1 Caesarean section?
4. Is this situation something you have encountered? If so, would you mind sharing your thoughts as you recall them at the time?
5. Do you think the hierarchical nature of clinical practice and medical school training plays a role here? Do you think it would be different in other settings?
