# Supplementary material for: Exploring the use of gender‐inclusive language amongst health care students and staff in obstetrics and gynaecology
Source: Med Educ. 2026 Apr 27;60(10):1110–20. doi: 10.1111/medu.70232 (PMC13569165; doi:10.1111/medu.70232)
Supplement: Supplementary file 2 [file MEDU-60-1110-s002.docx]

# **Supplementary Material 3**

**REFLEXIVITY STATEMENTS**

DA: I have dedicated much of my medical education career, including research, to social justice issues, particularly in supporting Sexual and Gender Minority (SGM) students and staff. As a queer, neurodivergent white woman with an unseen disability, my lived experiences, including navigating homophobic and ableist microaggressions, deeply inform my approach to interviewing and data interpretation. My previous roles as an SGM-identifying senior staff member in medical schools have further shaped my perspective. Throughout the research process, I remain mindful of how my identities and senior position may influence interactions and analyses.

GL: I am a doctoral research fellow on this project and a doctoral candidate in the Department of Education. My academic background lies in women’s, gender, and sexuality studies and its intersections with disability, race, and queerness. As a woman of colour with lived experiences of oppression, I am dedicated to improving equity for multiply marginalised communities through activism. As a relative newcomer to medical education research, this project has informed my understanding of the systemic barriers faced by SGM communities in medicine, particularly as I conducted several interviews and was exposed to a broad range of viewpoints on gender-inclusive language. I am interested in processes of transformational justice and social change through participatory, inclusive qualitative research methods.

JA: I am a gender-diverse doctor whose working week is predominantly in undergraduate medical education. I have my pronouns openly on my name badge/email signature, though also present in a traditionally masculine manner (with all the privileges that affords me alongside being white, non-disabled, middle-class etc). I am currently pleasantly confused about my gender, and this uncertainty informs how I approach questions of identity, language, and belonging. A good portion of my work involves EDI within my medical school, and I view open non-judgemental dialogue as a pathway to transformative learning, aligning with constructivist approaches to education. I have close friends and family whose perspectives on sex and gender differ significantly from my own (some of whom I am not out to) and have had a lot of practice engaging in compassionate, respectful disagreement. This influences my orientation to research, where I seek to foster understanding and identify common ground, while recognising that my approach may evolve over time.

MR: I am a white cis-woman who has experienced gender-diversity within my wider family, and this has informed my desire to improve my knowledge and understanding of how healthcare can support individuals. Feminist values shaped my upbringing and much of my career before medicine focussed on empowering marginalised groups and giving voices to those experience stigma and for whom society places barriers. During my formative years of medical education, conversations around gender identity were not commonplace, and therefore my views have been shaped by personal observations, witnessing social change and my experiences whilst training as a doctor. My experience within obstetrics and gynaecology has continued to shape my values regarding challenging discrimination within healthcare and upholding patient centred care, sensitive to individuals regardless of their identity. When conducting interviews, I continued to be mindful of my personal and professional experiences, and how these might shape my approach with participants.

BSSW: I approach this project as a qualitative researcher trained in education and design, with community-engaged work in India and current doctoral study at Oxford. Much of my scholarship asks how institutional language shapes belonging; that orientation drew my attention, during analysis, to how documentation, hierarchy, and teaching routines condition the uptake of gender-inclusive language. As an outsider to clinical medicine, I brought curiosity and distance, but I also risked importing assumptions from education. I addressed this by journalling reactions during reading/coding, inviting challenge from clinical co-authors, and revising interpretations when the data supported alternative accounts. My bilingual experience (English/Hindi) reminded me that terms can land differently across generations and institutional cultures. Throughout, I tried to hold two commitments together: care for gender-diverse patients and fairness to colleagues learning within constrained systems, keeping participants’ words foregrounded and my priors visible.

MA: I am a woman of colour working as a doctor on a training programme. This means that I frequently rotate onto different job roles and therefore am able to work with a great diversity of colleagues and patient groups. I have a keen interest in exploring the difficulties faced by marginal groups within healthcare. This is in part influenced by my own lived experiences of microaggressions but also the discrimination I have witnessed during my medical training towards individuals of marginalised groups. These experiences have shaped my commitment to promoting diversity, equity and inclusivity within healthcare. I recognise that my perspective and values as a woman of colour may differ greatly from those who do not have lived experience of such difficulties and therefore may influence my interpretation of certain responses from peers and colleagues. I therefore engage in reflection and discussion on topics related to diversity in healthcare and remain open to respectful discussion with those whose views, and lived experiences, may differ from mine.
