# Supplementary material for: Exploring the use of gender‐inclusive language amongst health care students and staff in obstetrics and gynaecology
Source: Med Educ. 2026 Apr 27;60(10):1110–20. doi: 10.1111/medu.70232 (PMC13569165; doi:10.1111/medu.70232)
Supplement: Supplementary file 3 [file MEDU-60-1110-s003.docx]

# **Supplementary Material 4**

**Themes, accompanying codes, and representative quotes**

| **Theme 1 – Generational Shifts** | ***Code 1: Power and Hierarchies*** | ***Code 2: Clinical, emotional, and training divides*** | ***Code 3: Structural issues in reporting concerns*** |
| --- | --- | --- | --- |
|  | “I think it becomes difficult when you're very junior and you don't have that, like, rapport with a consultant, which sometimes we don't have that rapport. And sometimes we know that the consultants are problematic for multiple reasons because they are, you know, might belong to a different generation. And you might, you know, they may carry other biases that you already know about.” (Angelina, Clinician) | “I think getting in at the ground floor, getting in in medical if you're learning about, you know, chests rather than breasts in medical school, then you're just going to carry on using that terminology, aren't you? It's like anything. If it's how you learnt it, then it's how you do it. You know, I do my caesareans in the way that I was taught, you know, 15, 16 years ago, even though people come through who do them differently.” (Reu, Clinician) | “I think it would be important to have a system where medical students can raise these concerns if there were a situation like that [where senior staff are discriminatory against SGM]. Sort of like a whistleblower way of doing it for medical students so that they can try to help without being negatively impacted. I don't know if there is a system for that, but yeah.” (Rosemary, Y5 medical student) |
| **Theme 2 – Language and Inclusivity** | ***Code 1: Discrimination and stigma*** | ***Code 2: Lived experience and inclusion*** | ***Code 3: ‘Clunky’ language changes*** |
|  | “I think in general, even doctors who might be very polite to patients to their face may have different opinions about the patients when they're discussing with other doctors. I think that's again, it's not black and white. There are some scenarios where there's obviously there's a big issue with patients being abusive towards healthcare staff as well, and doctors and healthcare staff need to vent that out to other staff members or counsellors too, in their own case. But I don’t think it's ever acceptable to be disrespectful about a patient, especially when they haven’t been disrespectful to you.” (Joseph, Y6 medical student) | “I know for the med school, we have medical badges that we can put our pronouns on, and a friend I have put gender neutral pronouns on theirs, and they no longer wear the badge because they said they just had people being like, ‘What? Why does it say they/them?’ and they just get confused or not be super good with it, and they changed years, and they just felt they didn't want to go into a whole new year group and almost have to come out to over 100 people.” (Linda, Y5 medical student) | “There's no more syllables between breastfeeding and chest feeding. There is no difference. It takes you 2 seconds to maybe adjust it if you've not been brought up with it, and I take a couple of seconds to adjust my brain as well. Like we're all – societally, we're brought up in this environment, but I don't think it's an excuse at all. ‘People who are pregnant’ is more inclusive and acknowledges the fact that pregnancies aren't determined by you being female or male in your gender or non-binary, whatever it might be, you can have a family.” (Rosemary, Y5 medical student) |
| **Theme 3- Clinical Practice and Education** | ***Code 1: Curricular gaps*** | ***Code 2: Clinical norms and behavioural changes*** | ***Code 3: Systemic barriers to accessing gender-inclusive care*** |
|  | “[Gender-inclusive language is] everyone's responsibility, to be honest, and you know, as we were saying, it has an impact on patient care. So, if medical schools aren't teaching it, then ultimately, they're training a group of doctors who are going to fail their patients. That's probably quite a blunt way of putting it, but that's how I see it.” (Rosemary, Y5 medical student) | “I think we have to give people an opportunity to get it wrong and correct themselves. I think and correct each other. I think it's fine to get it wrong if we're trying to get it right and you know, we acknowledge, you know, oops, I said she, but of course I meant he, fine ... I think it's very easy to make people feel like they're being told off. And if you do it publicly, you know, it becomes very hard to get people to change their behaviour.” (Judi, Clinician) | “I would also feel very helpless in like this outstanding referral, knowing that it can take years to be seen and this person is completely [un-helped] by their community care.” (Marie, Y5 medical student) |
| **Theme 4 – Intersectionality and Trans Rights** | ***Code 1: Perceived zero-sum conflict*** | ***Code 2: Politicisation and identity*** | ***Code 3: Individual versus systemic changes*** |
|  | “The hoopla over trans stuff takes away from other things that we should that, like takes away from the progress of other things, and it's just - I just really get really upset how people throw, like, if people don't like the trans issue, they throw away all the other progress that we've made in the last couple decades.” (Esme, Y1 midwifery student) | “It's so political. It's not just about an individual identity anymore. It's about like, there's this whole, like, TERF-yness [trans-exclusionary radical feminism] around it and that women's health is women's health, and that when you take away the woman from it, then you reduce the availability of gender, like that kind of inclusive space. I think it should be both. I think that we can have women and other gender identities included in all language.” (Marie, Y5 medical student) | *“*I hope that in, I don't know, 10 years, that all of this will be completely academic, and we've moved on, but as I guess, but women's space is still protected and there is still an acknowledgement that sex matters, especially for women, and some of the progress that we've made from that point of view isn't eroded, but there's no reason why it should be. But you never know with progress, there's unintended consequences.” (Joy, Clinician)” |
