# Supplementary material for: Exploring the use of gender‐inclusive language amongst health care students and staff in obstetrics and gynaecology
Source: Med Educ. 2026 Apr 27;60(10):1110–20. doi: 10.1111/medu.70232 (PMC13569165; doi:10.1111/medu.70232)
Supplement: Supplementary file 4 [file MEDU-60-1110-s004.docx]

# **Supplementary Material 2**

**INTERVIEW GUIDE**

**Interview Questions**

1. What does gender inclusive language mean to you?

2. Have you noticed any changes in the way gender inclusive language has been used in O&G in recent years?

3. Should gender-inclusive language be implemented in O&G in future? If so, how? If not, why?

4. What role does LGBTQ+ healthcare training have to play in healthcare education? Are there any links between such training and the use of gender-inclusive language in O&G?

5. Do you feel there is a difference between the way sexuality and gender diversity are approached in healthcare settings?

**Scenario Prompts**

***(Ask at least one – if time, ask two)***

**Case 1:**

You are an F1 doctor on a GP placement. A 22-year-old, James, has recently moved to the area and comes to see you relating to painful periods. He also asks if you can help him by writing to the gender identity services on his behalf. He was referred 14 months prior by his old GP for consideration of hormonal gender-affirming treatment but is still on a waiting list. He confides in you that he is finding his menstruation to be very distressing and has no idea what kind of things can be done to help.

a) How does this situation make you feel? What kind of attitudes may affect what a doctor does in this situation?
b) What stereotypes about trans or non-binary people might we be in danger of falling into?
c) Would you feel any different if this was someone asking us to write a letter for a different reason - for example with a physical disability asking us to chase social services for an occupational health assessment?
d) If you do feel differently - can you think why that might be?

**Case 2:**

You are a 4th Year Medical Student on your surgical placement. Today, you are in the clinic with the consultant, and you have been asked to take a history from Chris Briggs. When you call Chris into the clinic room, they are dressed androgynously, and you feel you would not know which sex they were born as.

a) Do you think it is necessary to discern the patient’s gender identity? Try to explain your reasoning.
b) How could you discern the patient’s gender identity?
c) How might perceptions and stereotypes be impacting things here? How might they impact Chris’ care?

You take the history from Chris and present it to your consultant. When the consultant asks you follow up questions, they refer to Chris as “she” and “her – you notice Chris looks uncomfortable when these terms are used but does not say anything.

d) How do you think Chris might feel?
e) What would you do?

**Definition of gender-inclusive language (if participants ask)**

To use gender-inclusive language is to recognise the spectrum of genders and identities that exist outside of a cisgender, male/female sex binary; for example, acknowledging that queer, trans, intersex, gender-diverse, and non-binary people may identify differently from their sex assigned at birth. Gender-inclusive language can look like asking and respecting one’s pronouns (she/her, he/him, they/them, any pronouns) when referring or speaking to someone; not assuming someone’s gender based solely on their outward presentation, and broadening our understanding on how gender is experienced and adjusting our language accordingly – for example, using terms like ‘pregnant people’ instead of ‘pregnant woman’.
